# Electroacupuncture at Hua Tuo Jia Ji Acupoints Reduced Neuropathic Pain and Increased GABA_A_ Receptors in Rat Spinal Cord

**DOI:** 10.1155/2018/8041820

**Published:** 2018-07-04

**Authors:** Siao-Wei Jiang, Yi-Wen Lin, Ching-Liang Hsieh

**Affiliations:** ^1^Master's Program for Traditional Chinese Veterinary Medicine, China Medical University, Taichung 40402, Taiwan; ^2^Graduate Institute of Acupuncture Science, College of Chinese Medicine, China Medical University, Taichung 40402, Taiwan; ^3^Chinese Medicine Research Center, China Medical University, Taichung 40402, Taiwan; ^4^Graduate Institute of Integrated Medicine, College of Chinese Medicine, China Medical University, Taichung 40402, Taiwan; ^5^Department of Chinese Medicine, China Medical University Hospital, Taichung 40447, Taiwan

## Abstract

Chronic constriction injury- (CCI-) induced neuropathic pain is the most similar model to hyperalgesia in clinical observation. Neuropathic pain is a neuronal dysfunction in the somatosensory system that may lead to spontaneous pain. In this study, electroacupuncture (EA) was applied at bilateral L4 and L6 of Hua Tuo Jia Ji points (EX-B2) for relieving neuropathic pain in rats. Eighteen Sprague-Dawley rats were randomly assigned to three groups: sham, 2-Hz EA, and 15-Hz EA groups. Following von Frey and cold plate tests, both the 2- and the 15-Hz EA groups had significantly lower mechanical and thermal hyperalgesia than the sham group. Western blot analysis results showed that *γ*-aminobutyric acid A (GABA_A_), adenosine A1 receptor (A1R), transient receptor potential cation channel subfamily V member 1 (TRPV1), TRPV4, and metabotropic glutamate receptor 3 (mGluR3) were similar in the dorsal root ganglion of all three groups. Furthermore, levels of GABA_A_ receptors were higher in the spinal cord of rats in the 2- and 15-Hz EA groups compared with the sham control group. This was not observed for A1R, TRPV1, TRPV4, or mGluR3 receptors. In addition, all the aforementioned receptors were unchanged in the somatosensory cortex of the study rats, suggesting a central spinal effect. The study results provide evidence to support the clinical use of EA for specifically alleviating neuropathic pain.

## 1. Introduction

According to the International Association for the Study of Pain, neuropathic pain is defined as pain caused by a lesion or disease of the somatosensory system [1]. Pain resulting from neuropathic pain is always accompanied by depression, anxiety, and sleep disturbance [2, 3]. Neuropathic pain can be further subdivided into peripheral and central neuropathy. Peripheral neuropathy includes painful diabetic neuropathy, human immunodeficiency virus-associated neuropathy, hemotherapy-induced peripheral neuropathy, and postherpetic neuralgia. By contrast, central neuropathy includes spinal injury, central poststroke pain, and compressive myelopathy among other conditions. Unbalanced neurotransmitters or neuromodulators always mismatch painful sensory inputs, which results in the generation of spontaneous painful sensations. In neuropathic pain, spontaneous mechanical or thermal hyperalgesia is often observed, and several central sensitizations are induced in the dorsal horn of the spinal cord (SC), such as sodium channels,* N*-methyl-d-aspartate (NMDA), *γ*-aminobutyric acid (GABA), and opioid receptors [4]. Medicines such as aspirin, acetaminophen, nonsteroidal anti-inflammation drugs, antidepressants, and opiates are currently used to treat neuropathic pain with limited success and side effects [5].

Adenosine is released from presynaptic terminals to bind postsynaptic A1, A2_A_, A2_B_, and A3 receptors for cardiovascular, immune, and nerve functions [6, 7]. Adenosine A1 receptor (A1R) is located mainly in peripheral sensory terminal [8], SC [9], and glial cells [10, 11]. Activation of A1R produced an analgesic effect in inflammatory and neuropathic pain models [12, 13]. A1R is reported to activate Gi protein and inhibit the cyclic adenosine monophosphate-protein kinase A pathway [14]. Injection of adenosine can reliably attenuate allodynia and hyperalgesia in many pain symptoms [7, 15]. However, several side effects result from activation of adenosine A2 receptor (A2R). It may induce vasodilation, a reduced heart rate, and severe cardiovascular obstacles.

GABA receptors are the main inhibitory neurotransmitters in the mammalian central nervous system. GABA receptors can be further subdivided into GABA_A_, GABA_B_, and GABA_C_. GABA_A_ is an ion channel that mediates fast inhibitory synaptic transmission and induces an influx of chloride, leading to stability of the neuronal membrane. Several factors can activate GABA_A_ for reducing neuronal excitability, and these are often used for anesthesia and pain management. Injection of GABA agonists is considered to be the most effective method of reducing pain signaling [16, 17]. In neuropathic pain, GABAergic interneurons initiate apoptosis via the caspase-3 pathway [18]. In addition, after nerve injury, GABA and glutamate decarboxylase 65 have been reported to be lower [19, 20].

The transient receptor potential (TRP) is a nonselective ion channel that can be activated following a tissue injury [21]. Transient receptor potential cation channel subfamily V member 1 (TRPV1) exists in nociceptors and the SC dorsal horn for pain signaling [22, 23]. TRPV1 can be activated by capsaicin, noxious heat (more than 43°C), and mechanical sensation [24–26]. TRPV1 is highly expressed in small C-fiber dorsal root ganglion (DRG) neurons and trigeminal and nodose ganglia [27]. Activation of TRPV1 induces sodium and calcium influx for neuronal depolarization [28, 29]. Depletion of TRPV1 gene results in insensitivity to noxious heat, radial heat, and hot-plate tests [30]. Inflammatory-material-induced thermal hyperalgesia was attenuated in TRPV1 knockout mice, suggesting the crucial role of TRPV1 in thermal pain sensation [31].

TRPV4 is a polymodal receptor that acts as an osmotic, mechanical, and thermal receptor [32]. Activation of TRPV4 releases calcitonin gene-related peptide and substance P into the SC dorsal horn [33]. TRPV4 is widely expressed in hair cells, kidney, lung, and peripheral sensory ganglia [34]. TRPV4 expressed in heterologous systems is usually activated by osmotic stimuli, resulting in cell swelling [35]. TRPV4 may also participate in the reduction of visceral pain [32]. Coexpression of TRPV1 and TRPV4 may synergistically play a role in nociception [36].

Neuropathic pain from surgery may damage c- and A*δ* fibers to increase the release of glutamate. Glutamate is a major excitatory neurotransmitter in the mammalian central nervous system that is released from presynaptic terminals for binding on four receptor subtypes: *α*-amino-3-hydroxy-5-methyl-4-isoxazolepropionic acid, NMDA, KA, and metabotropic glutamate receptor (mGluR) [37]. mGluR was indicated to be involved in neuropathic pain [38].

Acupuncture has been used for treating diseases for thousands of years. Increasing evidence suggests that electroacupuncture (EA) can be used to treat learning and memory impairment in ischemia rats [39], epilepsy [40], body weight control [41], and pain [42, 43]. Accordingly, the aim of the present study was to identify whether EA applied at Hua Tuo Jia Ji acupoints could reduce chronic constriction injury- (CCI-) induced neuropathic pain in rat models. We further investigated whether A1R, GABA_A_, TRPV1, TRPV4, and mGluR3 participate in the EA analgesic effect by evaluating DRG, SC, and somatosensory cortex (SSC) levels.

## 2. Materials and Methods

### 2.1. Animals

Eighteen male Sprague-Dawley rats weighing 201–250 g were purchased from BioLASCO (BioLASCO Taiwan Co., Ltd) and housed in the animal center of China Medical University (CMU). A 12-12-h light-dark cycle was maintained, and the room temperature was controlled at 25°C. Adequate food and water were provided. The Animal Care and Use Committee of CMU approved the use of these animals. In addition, all procedures were performed according to the* Guide for the Use of Laboratory Animals* (National Academy Press). The CCI model is often used to mimic clinical neuropathic pain by “tying 4-0 chromic gut sutures” in the sciatic nerve with the phenotype of hyperalgesia and allodynia. CCI was induced on the right sciatic nerve of rats, and their pain behavior was tested on day 5 to ensure the establishment of neuropathic pain. The sample size required an effect size of 0.6 in withdrawal threshold and an alpha of 0.05, and a power of 80%  was estimated to be 6 animals per group. Rats were randomly subdivided into three groups (*n* = 6/group): (1) sham, (2) 2-Hz EA, and (3) 15-Hz EA groups. Rats were anesthetized with 3%  isoflurane, and their right sciatic nerve was exposed. Furthermore, the nerve proximal to the trifurcation was ligated using four 4-0 chromic gut sutures. The surgical site was closed immediately using silk line before the rats were placed back in their cage.

### 2.2. EA Treatment

EA was applied at days 7, 8, and 9 using stainless steel needles (0.5′′, 32 G, Yu-Kuang, Taiwan) inserted into Hua Tuo Jia Ji acupoints at a depth of 2-3 mm, 5 mm from the spine at L4 and L6. EA was administered for 20 min immediately after the neuropathic injection. A stimulator (Trio 300, Ito, Japan) delivered 100-*μ*s square pulses of 2 mA for 15 min at 2 or 15 Hz. For the sham control group, we only inserted the needle and connected the animals to the stimulator without current input.

### 2.3. Animal Behavior

The von Frey test was adopted to examine mechanical hyperalgesia (IITC; Life Science Inc., USA). Rats were placed on a metal mesh and stimulated by applying a thin filament at the hind paw. The forces were recorded automatically when the rats withdrew their right hind paw. The cold plate test was used to assess thermal hyperalgesia during which the rats were placed on a cold plate apparatus (Panlab, Spain), with the temperature at 4°C. The total number of foot lifts was counted (right hind paw) for 5 min. The laboratory workers kept blind to treatment allocation during the experiments and analysis.

### 2.4. Western Blot Analysis

Rat DRG, SC, and SSC were immediately excised at day 9 after behavior test for protein extraction. We followed the methods of Liao et al. 2016 [40]. Total protein was prepared by homogenizing the hippocampi for 1 h at 4°C in a lysis buffer containing 20 mmol/L imidazole-HCl (pH 6.8), 100 mmol/L KCl, 2 mmol/L MgCl_2_, 20 mmol/L ethylene glycol tetraacetic acid (pH 7.0), 300 mmol/L sucrose, 1 mmol/L NaF, 1 mmol/L sodium vanadate, 1 mmol/L sodium molybdate, 0.2%  Triton X-100, and a proteinase inhibitor cocktail. From each sample, 30 *μ*g of protein was extracted and analyzed through a bicinchoninic acid protein assay. The extracted protein was subjected to 10%–15%  sodium dodecyl sulfate-Tris-glycine gel electrophoresis and transferred onto a nitrocellulose membrane. The membrane was blocked with 5%  nonfat milk in a TBST buffer (10 mmol/L Tris-buffered saline, pH 7.5; 100 mmol/L NaCl; and 0.1%  Tween 20) and incubated overnight at 4°C with the anti-GABA_A_ antibody (1:1000, Alomone, Israel), anti-A1R antibody (1:1000, Alomone), anti-TRPV1 (1:1000, Alomone), anti-TRPV4 antibody (1:1000, Alomone), and anti-GluR3 antibody (1:1000, Alomone) in TBST containing bovine serum albumin. Peroxidase-conjugated antibody (1:500) was used as the secondary antibody. The membrane was assessed using the ECL-Plus protein detection kit.

### 2.5. Statistical Analysis

All data are presented as mean ± standard error. Statistically significant differences among the sham, 2-Hz EA, and 15-Hz EA groups were analyzed through one-way analysis of variance, followed by Tukey's* post hoc *test. A* p* value less than 0.05 was considered statistically significant.

## 3. Results

**Figure 1:**
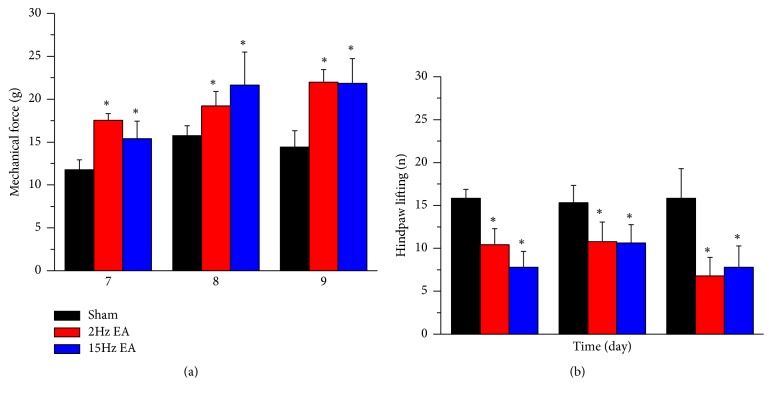
Withdrawal thresholds and hind paw lifting of rats in the sham (*n* = 6), 2-Hz EA (*n* = 6), and 15-Hz EA (*n* = 6) groups (*N* = 18) after CCI induction at days 7 to 9. (a) Mechanical forces of neuropathic pain rats on days 7 (7), 8 (8), and 9 (9). (b) Hind paw lifting of neuropathic pain rats on days 7 (7), 8 (8), and 9 (9). *∗p *< 0.01 versus sham group. CCI: chronic constriction injury; EA: electroacupuncture.

We first used the von Frey test to ensure the induction of CCI-induced mechanical hyperalgesia. Our data showed that the CCI had initiated mechanical hyperalgesia from day 7 to day 9 after induction in the sham group (Figure 1(a); 11.77 ± 1.16 g, 15.76 ± 1.15 g, 14.4 ± 1.92 g, respectively). We next discovered that the 2-Hz EA group had significantly lower mechanical hyperalgesia than the sham group (Figure 1(a); 17.53 ± 0.79 g, 19.21 ± 1.69 g, 21.98 ± 1.47 g, respectively). Similar results were also observed in the 15-Hz EA group (Figure 1(a); 15.39 ± 2.04 g, 21.63 ± 3.86 g, 21.84 ± 2.89 g, respectively). Next, we performed the cold plate test to determine whether thermal hyperalgesia was involved in the CCI-initiated neuropathic pain. Our results indicated that CCI reliably induced thermal hyperalgesia from day 7 to day 9 in the sham group (Figure 1(b); number of hind paw lifts: 15.83 ± 1.05, 15.33 ± 2.01, 15.83 ± 3.44, respectively). In both the 2-Hz group (Figure 1(b); 10.4 ± 1.9, 10.8 ± 2.28, 6.8 ± 2.13) and 15-Hz group (Figure 1(b); 7.8 ± 1.85, 10.6 ± 2.18, 7.8 ± 2.48), EA successfully attenuated thermal hyperalgesia. Accordingly, we suggest that 2- and 15-Hz EA can reliably attenuate both mechanical and thermal hyperalgesia.

**Figure 2:**
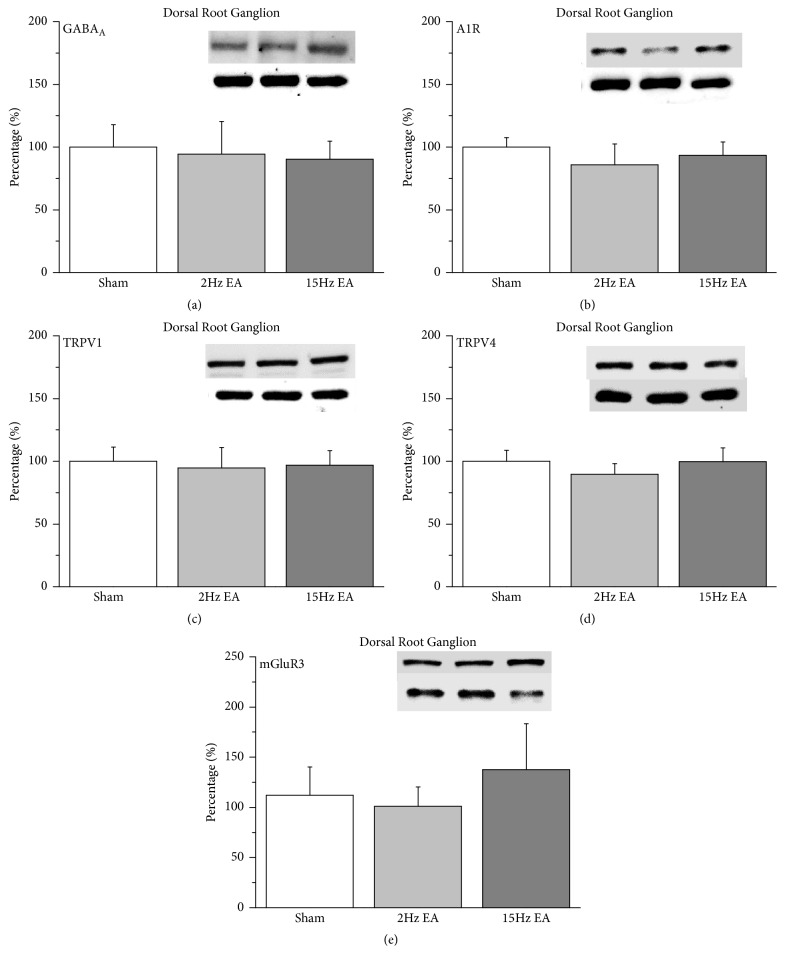
Expression levels of GABA_A_, A1R, TRPV1, TRPV4, and mGluR3 receptors in rat DRG. (a) GABA_A_, (b) A1R, (c) TRPV1, (d) TRPV4, and (e) mGluR3 expression levels in DRG from the sham, 2-Hz EA, and 15-Hz EA groups (from left to right). Sham: neuropathic pain rats with sham EA; 2 Hz: neuropathic pain rats that received 2-Hz EA; 15 Hz: neuropathic pain rats that received 15-Hz EA. *∗p *< 0.05 compared with the sham group. The Western blot bands at the top of each panel show the target protein. The lower bands are internal controls (*β*-actin). A1R: adenosine A1 receptor; DRG: dorsal root ganglion; EA: electroacupuncture; GABA: *γ*-aminobutyric acid; TRPV1: transient receptor potential cation channel subfamily V member 1; mGluR3: metabotropic glutamate receptor 3; TRPV4: transient receptor potential cation channel subfamily V member 4.

Western blot analysis was performed to clarify the involvement of receptors in the EA analgesia of CCI-induced neuropathic pain. Our results indicated that the protein levels of GABA_A_ receptors in rat DRG were not changed in the sham, 2-Hz EA, and 15-Hz EA groups (Figure 2(a); 100.01%  ± 17.97%, 94.44%  ± 25.91%, and 90.17%  ± 14.62%, respectively;* p* > 0.05,* n* = 6). We next verified the effect of analgesic A1R on peripheral DRG neurons. We determined that there was no difference among the sham, 2-Hz EA, and 15-Hz EA groups (Figure 2(b); 100.01%  ± 7.6%, 86.01%  ± 16.43%, and 93.51%  ± 10.61%, respectively;* p* > 0.05,* n* = 6). The phenomenon was also observed for TRPV1 (Figure 2(c); 100.01%  ± 11.15%, 94.61%  ± 16.50%, and 96.81%  ± 11.53%;* p* > 0.05,* n* = 6), TRPV4 (Figure 2(d); 100.01%  ± 8.66%, 89.68%  ± 8.41%, and 99.81%  ± 10.95%;* p* > 0.05,* n* = 6), and mGluR3 (Figure 2(e); 100.04%  ± 28.14%, 101.16%  ± 19.23%, and 137.52%  ± 45.89%;* p* > 0.05,* n* = 6) expression in rat DRG.

**Figure 3:**
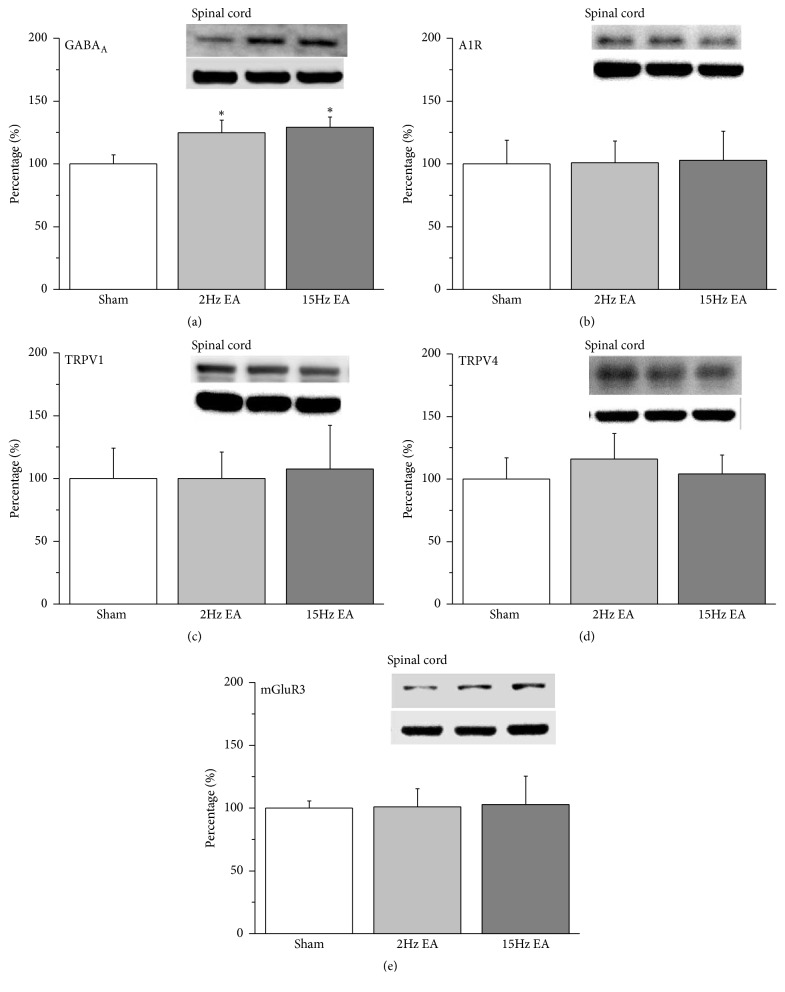
Expression levels of GABA_A_, A1R, TRPV1, TRPV4, and mGluR3 receptors in rat SC. (a) GABA_A_, (b) A1R, (c) TRPV1, (d) TRPV4, and (e) mGluR3 expression levels in the SC of the rats from the sham, 2-Hz EA, and 15-Hz EA groups (from left to right). Sham: neuropathic pain rats with sham EA; 2 Hz: neuropathic pain rats that received 2-Hz EA; 15 Hz: neuropathic pain rats that received 15-Hz EA. *∗p *< 0.05 compared with the sham group. The Western blot bands at the top of each panel show the target protein. The lower bands are internal controls (*β*-actin). A1R: adenosine A1 receptor; DRG: dorsal root ganglion; EA: electroacupuncture; GABA: *γ*-aminobutyric acid; TRPV1: transient receptor potential cation channel subfamily V member 1; mGluR3: metabotropic glutamate receptor 3; TRPV4: transient receptor potential cation channel subfamily V member 4.

We further analyzed the levels of the aforementioned receptors in the central SC. Our data demonstrated that both 2- and 15-Hz EA significantly increased the protein levels of GABA_A_ receptors in rat SC (Figure 3(a); 124.79%  ± 9.86%  and 129.29%  ± 8.03%;* p* < 0.05,* n* = 6). We then evaluated whether analgesic A1R also participated in the EA process. Our results demonstrated that the potentiation was not observed in either the 2- or the 15-Hz EA group (Figure 3(b); 101.08%  ± 17.19% and 102.87%  ± 23.1%;* p* > 0.05,* n* = 6). This phenomenon was also not observed in TRPV1 (Figure 3(c); 100.09% ± 20.92%  and 107.64 ± 34.72;* p* > 0.05,* n* = 6), TRPV4 (Figure 3(d); 115.83% ± 20.39%  and 104.05%  ± 14.97%;* p* > 0.05,* n* = 6), or mGluR3 (Figure 3(e); 101.08%  ± 14.34%  and 102.87%  ± 22.65%;* p* > 0.05,* n* = 6) expression in SC.

**Figure 4:**
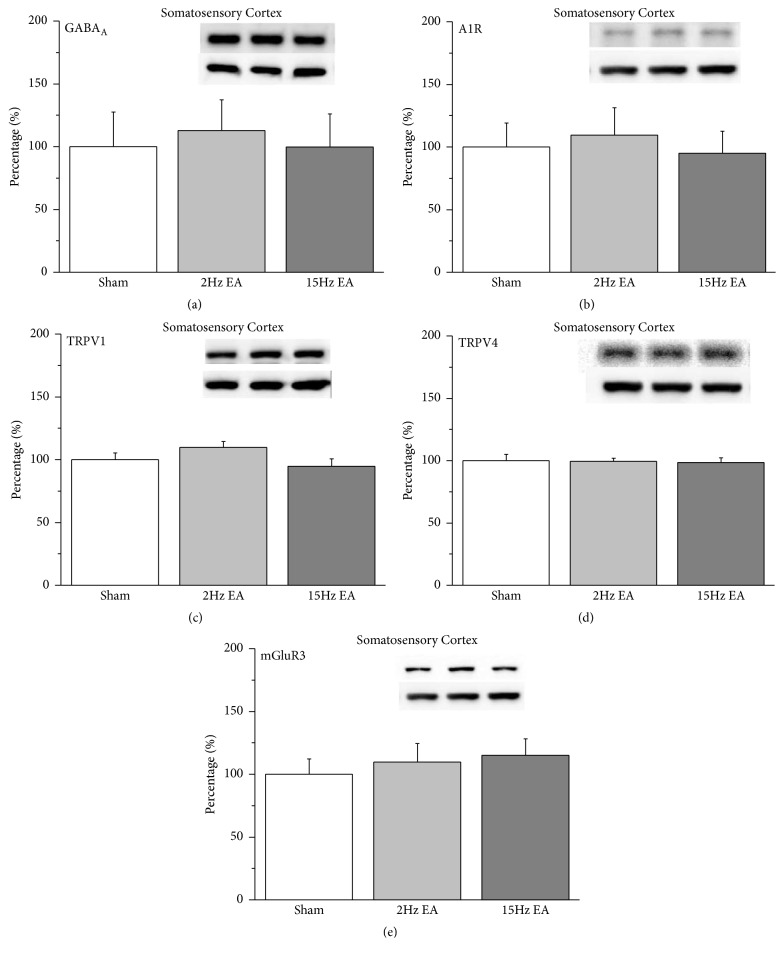
Expression levels of GABA_A_, A1R, TRPV1, TRPV4, and mGluR3 receptors in rat SSC. (a) GABA_A_, (b) A1R, (c) TRPV1, (d) TRPV4, and (e) mGluR3 expression levels in the SSC of rats from the sham, 2-Hz EA, and 15-Hz EA groups (from left to right). Sham: neuropathic pain rats with sham EA; 2 Hz: neuropathic pain rats that received 2-Hz EA; 15 Hz: neuropathic pain rats that received 15-Hz EA. *∗p *< 0.05 compared with the sham group. The Western blot bands at the top of each panel show the target protein. The lower bands are internal controls (*β*-actin). A1R: adenosine A1 receptor; DRG: dorsal root ganglion; EA: electroacupuncture; GABA: *γ*-aminobutyric acid; TRPV1: transient receptor potential cation channel subfamily V member 1; mGluR3: metabotropic glutamate receptor 3; TRPV4: transient receptor potential cation channel subfamily V member 4.

Furthermore, we further tested whether these receptors were involved in EA analgesia in the rat SSC. Neither 2- nor 15-Hz EA altered the protein levels of GABA_A_ receptors in rat SSC (Figure 4(a); 112.84%  ± 24.48%  and 99.81%  ± 26.06%;* p* > 0.05,* n* = 6). We next showed that A1R did not participate in EA analgesia because the protein level was not altered in either the 2- or the 15-Hz EA group (Figure 4(b); 109.28%  ± 22.05%  and 95.05%  ± 17.43%;* p* > 0.05,* n* = 6). Protein levels remained unchanged in TRPV1 (Figure 4(c); 109.84%  ± 4.55%  and 94.56%  ± 6.13%;* p* > 0.05,* n* = 6), TRPV4 (Figure 4(d); 99.5%  ± 2.23%  and 98.48%  ± 3.76%;* p* > 0.05,* n* = 6), and mGluR3 (Figure 4(e); 109.73% ± 14.87%  and 114.98% ± 13.38%;* p* > 0.05,* n* = 6) expressions in the rat cortex.

## 4. Discussion

EA has been widely used for pain relief. A recent study [44] indicated that high-frequency (100-Hz) EA obtained superior results to low-frequency (2-Hz) EA in the rat ankle sprain pain model. EA can relieve acute pain by releasing opiates to activate *μ*-, *δ*-, and *κ*-opioid receptors. By contrast, EA can regulate persistent pain by activating *μ*- and *δ*-opioid receptors [45]. Abuaisha et al. [46] suggested that acupuncture is a potential treatment for chronic painful peripheral diabetic neuropathy. Cruccu et al. [47] stated that pharmacological relief of neuropathic pain is always insufficient. Electrical neurostimulation is therapeutic in chronic neuropathic pains. One study suggested that EA is more favorable than high-frequency transcutaneous electrical nerve stimulation. Our previous study [48] demonstrated that EA at Zusanli and Shangjuxu acupoints significantly reduced both mechanical and thermal hyperalgesia by reducing cerebral TRPV4 expressions but not TRPV1 expression.

EA at 2 Hz can reduce pain depending on the noradrenergic descending pathway and spinal GABAergic modulation. By contrast, high-frequency EA at 100 Hz mainly acts on GABA_B_ mechanisms [49]. EA at 2 Hz can reliably decrease pain signaling through GABA_A_ in the dorsal anterior pretectal nucleus. Furthermore, 100-Hz EA can attenuate pain via *μ*-opioid and 5-hydroxytryptamine 1 (5-HT1) receptors in the ventral anterior pretectal nucleus [49]. Fusumada et al. [50] suggested that EA at the ST36 acupoint may regulate GABAergic transmission on descending pathways in periaqueductal areas. Injection of a GABA receptor agonist, gabazine or saclofen, attenuated the therapeutic effect of EA on cold allodynia in rats. These results indicate the crucial role of both GABA_A_ and GABA_B_ receptors in neuropathic rats at spinal levels [51]. Xing et al. [52] demonstrated that neuronal hyperactivity in spinal pain transmission was enhanced after nerve injury and further developed into neuropathic pain. EA at 2 Hz was curative in neuropathic pain with the expression of LTD in the C-fiber in SNL rats. The phenomenon could be blocked by NMDA and an opioid receptor antagonist. By contrast, 100-Hz EA induced LTP in SNL rats, which was mainly mediated by endogenous GABAergic and serotonergic inhibitory systems [52]. In this study, EA administered at Hua Tuo Jia Ji acupoints reduced neuropathic pain by increasing the protein level of GABA_A_ receptor in the SC.

Goldman et al. [53] determined that adenosine was released during manual acupuncture to activate adenosine A1 but not A2 receptors. They also showed that direct injection of A1R agonist could mimic the analgesic effect of acupuncture. Inhibition of adenosine-degradation enzymes enhanced the concentration of adenosine that was positively related to pain relief [53]. Acupuncture and adenosine agonist injected into Zusanli acupoints significantly reduced electrical pain signals in the anterior cingulate cortex. The phenomenon was majorly mediated by the activation of A1R but not A2R. A recent study [54] also showed that interstitial adenosine concentration was increased through acupuncture at the ST36 acupoint for 30 minutes. The phenomenon was only observed at acupoints with rotation, but not when the needle was delivered without rotation or with rotation at a nonacupoint. Intrathecal injection of adenosine reduced spontaneous pain in humans with neuropathic pain [55]. By contrast, A1R but not A2R activation at the spinal level attenuated spontaneous pain [56]. Our data indicated that A1R receptor was unaltered when EA was delivered at Hua Tuo Jia Ji acupoints. Thus, we suggest that A1R is crucial for local analgesia.

TRPV1 was increased in a complete Freund's adjuvant-induced inflammatory pain model from day 1 to day 21. Subcutaneous or intrathecal injection of TRPV1 antagonist capsazepine attenuated thermal hyperalgesia in an inflammatory pain model [57]. Wu et al. [58] indicated that injection of capsaicin, a TRPV1 agonist, into the ST36 acupoint relieved inflammatory pain. This suggested that capsaicin replicated the analgesic effect of acupuncture [58]. TRPV4 participated in inflammatory thermal and mechanical hyperalgesia [59]. Chemotherapy-induced neuropathic pain was ameliorated by TRPV4 antisense oligodeoxynucleotide [60]. TRPV4 plays an important role in several pain syndromes such as acute inflammatory, chronic, neuropathic, and chemotherapy-mediated pain. Chen et al. [61] indicated that EA at bilateral ST36 acupoints reduced the potentiation of TRPV1 and TRPV4 in mice with inflammatory pain. Another study [38] demonstrated that activation of mGluRs attenuated glutamatergic input, which explains the analgesic effect of its agonists on neuropathic pain. Chiechio et al. [62] suggested that activation of mGlu2/3 could reduce neuropathic pain [62]. Our data showed that TRPV1, TRPV4, and mGluR3 were not altered by EA performed at Hua Tuo Jia Ji acupoints.

In summary, we conclude that EA at bilateral L4 and L6 of Hua Tuo Jia Ji acupoints significantly reduces rat neuropathic pain, as determined by measuring mechanical and thermal hyperalgesia through von Frey and cold plate tests. We also determined that spinal GABA_A_ receptor—but not A1R, TRPV1, TRPV4, or mGluR3—responded to EA analgesia.

## References

[1] Jensen T. S., Baron R., Haanpää M. (2011). A new definition of neuropathic pain. *PAIN*.

[2] Cheng J., Long J., Hui X., Lei D., Zhang H. (2017). Effects of microvascular decompression on depression and anxiety in trigeminal neuralgia: A prospective cohort study focused on risk factors and prognosis. *Clinical Neurology and Neurosurgery*.

[3] Zhu C., Xu Q., Wang C., Mao Z., Lin N. (2017). Evidence that CA3 is underling the comorbidity between pain and depression and the co-curation by wu-tou decoction in neuropathic pain. *Scientific Reports*.

[4] Dickenson A. H., Chapman V., Green G. M. (1997). The pharmacology of excitatory and inhibitory amino acid-mediated events in the transmission and modulation of pain in the spinal cord. *General Pharmacology: The Vascular System*.

[5] Kroenke K., Krebs E. E., Bair M. J. (2009). Pharmacotherapy of chronic pain: a synthesis of recommendations from systematic reviews. *General Hospital Psychiatry*.

[6] Gessi S., Merighi S., Fazzi D., Stefanelli A., Varani K., Borea P. A. (2011). Adenosine receptor targeting in health and disease. *Expert Opinion on Investigational Drugs*.

[7] Sawynok J. (2015). Adenosine receptor targets for pain. *Neuroscience*.

[8] Lima F. O., Souza G. R., Verri W. A. (2010). Direct blockade of inflammatory hypernociception by peripheral A1 adenosine receptors: Involvement of the NO/cGMP/PKG/KATP signaling pathway. *PAIN*.

[9] Ackley M. A., Governo R. J. M., Cass C. E., Young J. D., Baldwin S. A., King A. E. (2003). Control of glutamatergic neurotransmission in the rat spinal dorsal horn by the nucleoside transporter ENT1. *The Journal of Physiology*.

[10] Boison D., Chen J.-F., Fredholm B. B. (2010). Adenosine signaling and function in glial cells. *Cell Death & Differentiation*.

[11] Magni G., Ceruti S. (2014). The purinergic system and glial cells: Emerging costars in nociception. *BioMed Research International*.

[12] Dickenson A. H., Stanfa L. C., Kontinen V., Suzuki R., Carpenter K. (2000). Comment on Svendsen et al., some problems with wind-up and its calculation, PAIN 83 (1999) 109-111. *PAIN*.

[13] Sawynok J. (1998). Adenosine receptor activation and nociception. *European Journal of Pharmacology*.

[14] Chen J.-F., Lee C.-F., Chern Y. (2014). Adenosine receptor neurobiology: overview. *International Review of Neurobiology*.

[15] Magni G., Riccio D., Ceruti S. (2017). Tackling chronic pain and inflammation through the purinergic system. *Current Medicinal Chemistry*.

[16] Ang S. T., Lee A. T. H., Foo F. C., Ng L., Low C.-M., Khanna S. (2015). GABAergic neurons of the medial septum play a nodal role in facilitation of nociception-induced affect. *Scientific Reports*.

[17] Zhang C., Chen R., Zhang Y. (2017). Reduced GABAergic transmission in the ventrobasal thalamus contributes to thermal hyperalgesia in chronic inflammatory pain. *Scientific Reports*.

[18] Scholz J., Broom D. C., Youn D.-H. (2005). Blocking caspase activity prevents transsynaptic neuronal apoptosis and the loss of inhibition in lamina II of the dorsal horn after peripheral nerve injury. *The Journal of Neuroscience*.

[19] Castro-Lopes J. M., Malcangio M., Pan B. H., Bowery N. G. (1995). Complex changes of GABAA and GABAB receptor binding in the spinal cord dorsal horn following peripheral inflammation or neurectomy. *Brain Research*.

[20] Castro-Lopes J., Tavares I., Coimbra A. (1993). GABA decreases in the spinal cord dorsal horn after peripheral neurectomy. *Brain Research*.

[21] Laing R. J., Dhaka A. (2016). ThermoTRPs and Pain. *The Neuroscientist*.

[22] Caterina M. J., Leffler A., Malmberg A. B. (2000). Impaired nociception and pain sensation in mice lacking the capsaicin receptor. *Science*.

[23] Davis J. B., Gray J., Gunthorpe M. J. (2000). Vanilloid receptor-1 is essential for inflammatory thermal hyperalgesia. *Nature*.

[24] Caterina M. J., Schumacher M. A., Tominaga M., Rosen T. A., Levine J. D., Julius D. (1997). The capsaicin receptor: a heat-activated ion channel in the pain pathway. *Nature*.

[25] Urano H., Ara T., Fujinami Y., Yukihiro Hiraoka B. (2012). Aberrant TRPV1 expression in heat hyperalgesia associated with trigeminal neuropathic pain. *International Journal of Medical Sciences*.

[26] Watabiki T., Kiso T., Kuramochi T. (2011). Amelioration of neuropathic pain by novel transient receptor potential vanilloid 1 antagonist AS1928370 in rats without hyperthermic effect. *The Journal of Pharmacology and Experimental Therapeutics*.

[27] Kobayashi K., Fukuoka T., Obata K. (2005). Distinct expression of TRPM8, TRPA1, and TRPV1 mRNAs in rat primary afferent neurons with A*δ*/C-fibers and colocalization with trk receptors. *Journal of Comparative Neurology*.

[28] Pecze L., Blum W., Schwaller B. (2013). Mechanism of capsaicin receptor TRPV1-mediated toxicity in pain-sensing neurons focusing on the effects of Na+/Ca2+ fluxes and the Ca2+-binding protein calretinin. *Biochimica et Biophysica Acta (BBA) - Molecular Cell Research*.

[29] Tsumura M., Sobhan U., Muramatsu T. (2012). TRPV1-mediated calcium signal couples with cannabinoid receptors and sodium-calcium exchangers in rat odontoblasts. *Cell Calcium*.

[30] Christoph T., Bahrenberg G., De Vry J. (2008). Investigation of TRPV1 loss-of-function phenotypes in transgenic shRNA expressing and knockout mice. *Molecular and Cellular Neuroscience*.

[31] Liao H.-Y., Hsieh C.-L., Huang C.-P., Lin Y.-W. (2017). Electroacupuncture attenuates CFA-induced inflammatory pain by suppressing Nav1.8 through S100B, TRPV1, opioid, and adenosine pathways in mice. *Scientific Reports*.

[32] Brierley S. M., Page A. J., Hughes P. A. (2008). Selective role for TRPV4 ion channels in visceral sensory pathways. *Gastroenterology*.

[33] Grant A. D., Cottrell G. S., Amadesi S. (2007). Protease-activated receptor 2 sensitizes the transient receptor potential vanilloid 4 ion channel to cause mechanical hyperalgesia in mice. *The Journal of Physiology*.

[34] White J. P. M., Cibelli M., Urban L., Nilius B., McGeown J. G., Nagy I. (2016). TRPV4: Molecular conductor of a diverse orchestra. *Physiological Reviews*.

[35] Strotmann R., Harteneck C., Nunnenmacher K., Schultz G., Plant T. D. (2000). OTRPC4, a nonselective cation channel that confers sensivity to extracellular osmolarity. *Nature Cell Biology*.

[36] Cao D.-S., Yu S.-Q., Premkumar L. S. (2009). Modulation of transient receptor potential vanilloid 4-mediated membrane currents and synaptic transmission by protein kinase C. *Molecular Pain*.

[37] Hollmann M., Heinemann S. (1994). Cloned glutamate receptors. *Annual Review of Neuroscience*.

[38] Zhang H.-M., Chen S.-R., Pan H.-L. (2009). Effects of activation of group III metabotropic glutamate receptors on spinal synaptic transmission in a rat model of neuropathic pain. *Neuroscience*.

[39] Kuo C.-T., Lin Y.-W., Tang N.-Y., Cheng C.-Y., Hsieh C.-L. (2016). Electric stimulation of the ears ameliorated learning and memory impairment in rats with cerebral ischemia-reperfusion injury. *Scientific Reports*.

[40] Liao E.-T., Tang N.-Y., Lin Y.-W., Liang Hsieh C. (2017). Long-term electrical stimulation at ear and electro-acupuncture at ST36-ST37 attenuated COX-2 in the CA1 of hippocampus in kainic acid-induced epileptic seizure rats. *Scientific Reports*.

[41] Choowanthanapakorn M., Lu K.-W., Yang J., Hsieh C.-L., Lin Y.-W. (2015). Targeting TRPV1 for Body Weight Control using TRPV1-/-Mice and Electroacupuncture. *Scientific Reports*.

[42] Lu K.-W., Hsu C.-K., Hsieh C.-L., Yang J., Lin Y.-W. (2016). Probing the effects and mechanisms of electroacupuncture at ipsilateral or contralateral ST36-ST37 acupoints on CFA-induced inflammatory pain. *Scientific Reports*.

[43] Yen L. T., Hsieh C. L., Hsu H. C., Lin Y. W. (2017). Targeting ASIC3 for relieving mice fibromyalgia pain: roles of electroacupuncture, opioid, and adenosine. *Scientific Reports*.

[44] Tae S. H. (2007). The effect of 2 Hz and 100 Hz electrical stimulation of acupoint on ankle sprain in rats. *Journal of Korean Medical Science*.

[45] Zhang R., Lao L., Ren K., Berman B. M. (2014). Mechanisms of acupuncture-electroacupuncture on persistent pain. *Anesthesiology*.

[46] Abuaisha B. B., Costanzi J. B., Boulton A. J. M. (1998). Acupuncture for the treatment of chronic painful peripheral diabetic neuropathy: a long-term study. *Diabetes Research and Clinical Practice*.

[47] Cruccu G., Aziz T. Z., Garcia-Larrea L. (2007). EFNS guidelines on neurostimulation therapy for neuropathic pain. *European Journal of Neurology*.

[48] Hsu H., Tang N., Lin Y., Li T., Liu H., Hsieh C. (2014). Effect of electroacupuncture on rats with chronic constriction injury-induced neuropathic pain. *The Scientific World Journal*.

[49] Silva J. R. T., Silva M. L., Prado W. A. (2011). Analgesia induced by 2- or 100-Hz electroacupuncture in the rat tail-flick test depends on the activation of different descending pain inhibitory mechanisms. *The Journal of Pain*.

[50] Zienowicz M., Wisłowska-Stanek A., Lehner M. (2007). Fluoxetine attenuates the effects of pentylenetetrazol on rat freezing behavior and c-Fos expression in the dorsomedial periaqueductal gray. *Neuroscience Letters*.

[51] Park J.-H., Han J.-B., Kim S.-K. (2010). Spinal GABA receptors mediate the suppressive effect of electroacupuncture on cold allodynia in rats. *Brain Research*.

[52] Xing G. G., Liu F. Y., Qu X. X., Han J. S., Wan Y. (2007). Long-term synaptic plasticity in the spinal dorsal horn and its modulation by electroacupuncture in rats with neuropathic pain. *Experimental Neurology*.

[53] Goldman N., Chen M., Fujita T. (2010). Adenosine A1 receptors mediate local anti-nociceptive effects of acupuncture. *Nature Neuroscience*.

[54] Takano T., Chen X., Luo F. (2012). Traditional acupuncture triggers a local increase in adenosine in human subjects. *The Journal of Pain*.

[55] Eisenach J. C., Rauck R. L., Curry R. (2003). Intrathecal, but not intravenous adenosine reduces allodynia in patients with neuropathic pain. *PAIN*.

[56] Zahn P. K., Straub H., Wenk M., Pogatzki-Zahn E. M. (2007). Adenosine A1 but not A2a receptor agonist reduces hyperalgesia caused by a surgical incision in rats: A pertussis toxin-sensitive G protein-dependent process. *Anesthesiology*.

[57] Luo H., Xu I. S., Chen Y. (2008). Behavioral and electrophysiological evidence for the differential functions of TRPV1 at early and late stages of chronic inflammatory nociception in rats. *Neurochemical Research*.

[58] Wu S.-Y., Chen W.-H., Hsieh C.-L., Lin Y.-W. (2014). Abundant expression and functional participation of TRPV1 at Zusanli acupoint (ST36) in mice: mechanosensitive TRPV1 as an “acupuncture-responding channel”. *BMC Complementary and Alternative Medicine*.

[59] Alessandri-Haber N., Dina O. A., Joseph E. K., Reichling D., Levine J. D. (2006). A transient receptor potential vanilloid 4-dependent mechanism of hyperalgesia is engaged by concerted action of inflammatory mediators. *The Journal of Neuroscience*.

[60] Alessandri-Haber N., Dina O. A., Yeh J. J., Parada C. A., Reichling D. B., Levine J. D. (2004). Transient Receptor Potential Vanilloid 4 Is Essential in Chemotherapy-Induced Neuropathic Pain in the Rat. *The Journal of Neuroscience*.

[61] Chen W.-H., Tzen J. T. C., Hsieh C. L. (2012). Attenuation of TRPV1 and TRPV4 expression and function in mouse inflammatory pain models using electroacupuncture. *Evidence-Based Complementary and Alternative Medicine*.

[62] Chiechio S., Copani A., Melchiorri D. (2004). Metabotropic receptors as targets for drugs of potential use in the treatment of neuropathic pain. *Journal of Endocrinological Investigation*.

